# Effects of Duodenal Infusion of Lauric Acid and L-Tryptophan, Alone and Combined, on Fasting Glucose, Insulin and Glucagon in Healthy Men

**DOI:** 10.3390/nu11112697

**Published:** 2019-11-07

**Authors:** Christina McVeay, Penelope C. E. Fitzgerald, Michael Horowitz, Christine Feinle-Bisset

**Affiliations:** Adelaide Medical School, Centre of Research Excellence in Translating Nutritional Science to Good Health, Level 5 Adelaide Health and Medical Sciences Building, Corner North Terrace and George Street, Adelaide 5005, Australia; christina.mcveay@adelaide.edu.au (C.M.); penelope.fitzgerald@adelaide.edu.au (P.C.E.F.); michael.horowitz@adelaide.edu.au (M.H.)

**Keywords:** amino acid, fatty acid, glycaemic control, blood glucose, humans

## Abstract

The fatty acid, lauric acid (‘C12’), and the amino acid, tryptophan (‘Trp’), when given intraduodenally at loads that individually do not affect energy intake, have recently been shown to stimulate plasma cholecystokinin, suppress ghrelin and reduce energy intake much more markedly when combined. Both fatty acids and amino acids stimulate insulin secretion by distinct mechanisms; fatty acids enhance glucose-stimulated insulin secretion, while amino acids may have a direct effect on pancreatic β cells. Therefore, it is possible that, by combining these nutrients, their effects to lower blood glucose may be enhanced. We have investigated the potential for the combination of C12 and Trp to have additive effects to reduce blood glucose. To address this question, plasma concentrations of glucose, insulin and glucagon were measured in 16 healthy, lean males during duodenal infusions of saline (control), C12 (0.3 kcal/min), Trp (0.1 kcal/min), or C12+Trp (0.4 kcal/min), for 90 min. Both C12 and C12+Trp moderately reduced plasma glucose compared with control (*p* < 0.05). C12+Trp, but not C12 or Trp, stimulated insulin and increased the insulin-to-glucose ratio (*p* < 0.05). There was no effect on plasma glucagon. In conclusion, combined intraduodenal administration of C12 and Trp reduced fasting glucose in healthy men, and this decrease was driven primarily by C12. The effects of these nutrients on postprandial blood glucose and elevated fasting blood glucose in type 2 diabetes warrant evaluation.

## 1. Introduction

Fat or protein, when consumed immediately before a carbohydrate-containing meal, may reduce the postprandial glycaemic excursion in health and type 2 diabetes substantially [1,2,3]. The slowing of gastric emptying and the release of gut hormones, particularly the incretin hormone, glucagon-like peptide-1 (GLP-1), are central to this effect [4,5]. The slowing of gastric emptying reflects, at least in part, the stimulation of pyloric contractile activity as a result of inhibitory feedback arising from the presence of nutrients in the small intestine [6]. There is considerable variation between nutrients in their effects to stimulate gut and glucoregulatory hormones; fat appears to be a more potent stimulant of GLP-1 than protein, whereas protein appears to have greater effects on insulin and glucagon [7,8]. Fatty acids enhance glucose-stimulated insulin secretion [9], while amino acids may have a direct effect on pancreatic β cells to simulate insulin release independent of glucose [10].

In this context, lauric acid, a fatty acid containing 12 carbon atoms (‘C12’), and tryptophan (‘Trp’), an aromatic amino acid, are of particular interest [11,12,13], since both potently stimulate pyloric contractile activity and gut hormones. For example, Trp, when administered intraduodenally at a dose of 0.15 kcal/min, but not 0.075 kcal/min, stimulated plasma insulin, glucagon and GLP-1 concentrations modestly, but had no effect on blood glucose in healthy males [11], while intragastric administration of 3 g, but not 1.5 g, of Trp attenuated the blood glucose response to a mixed-nutrient drink in lean and obese participants [13]. In people with type 2 diabetes, C12, when delivered to the distal small intestine in enteric-coated capsules prior to meals, lowered postprandial blood glucose and stimulated GLP-1, but did not stimulate insulin [14]. Accordingly, blood glucose lowering may reflect the stimulation of GLP-1 receptors on portal vagal afferents [15], to enhance hepatic and peripheral glucose uptake [16].

It is thought that C12 and Trp stimulate gut and glucoregulatory hormones via distinct populations of receptors located on enteroendocrine cells [17,18,19]; therefore, it would not be surprising if the combination of these nutrients would enhance their individual effects to lower blood glucose. In support of this hypothesis, C12 and Trp, when given intraduodenally at loads that individually do not affect energy intake, reduce energy intake substantially when combined, associated with much greater stimulation of cholecystokinin (CCK) and suppression of ghrelin, in healthy males [20]. In contrast, while C12 stimulated GLP-1, this effect was not augmented when C12 and Trp were combined. Given the important role of GLP-1 in blood glucose regulation, as discussed above, this latter finding suggests, however, that C12 and Trp, in contrast to their effects on energy intake, may not have additive effects to lower blood glucose.

We have now investigated the potential for the combination of C12 and Trp to reduce fasting blood glucose more than each nutrient individually. We assayed samples from our previous study to quantify plasma concentrations of glucose, insulin and glucagon [20].

## 2. Materials and Methods

### 2.1. Study Participants

As described in the primary publication [20], 16 healthy, lean mean (mean age: 24 ± 1.5 years; body mass index (BMI): 22.9 ± 0.4 kg/m²) were included. Participants were recruited, as described previously [20]. All participants had been weight-stable and were unrestrained eaters (score ≤12 on the eating restraint section (Factor 1) of the Three-Factor Eating Questionnaire [21]). None had a history of gastrointestinal (GI) symptoms or surgery, used supplements or medications known to affect GI function or appetite, smoked, consumed >20 g/d of alcohol, or were vegetarians. Once enrolled into the study, each participant was allocated a random treatment sequence based on balanced randomisation generated using an online tool (www.randomization.com), by an investigator who was not involved in data analysis (P.C.E.F.). Both the participant and the investigator who assessed outcomes (C.M.) were blinded to the randomisation. The Human Research Ethics Committee of the Central Adelaide Local Hospital Network approved the study protocol, and all participants provided informed, written consent prior to their inclusion. The study was registered as a clinical trial at the Australian and New Zealand Clinical Trial Registry (www.anzctr.org.au, trial number: ANZCTR 12613000899741).

### 2.2. Study Design and Protocol

Comprehensive information about the design of the study, which was a randomised, double-blind, cross-over design, including the participant enrolment flow diagram, has been published [20]. Briefly, each participant was studied on four separate days, 3–10 days apart, to evaluate the effect of intraduodenal administration of (1) lauric acid (‘C12’; load: 27 kcal, 0.3 kcal/min), (2) L-tryptophan (‘Trp’; load: 9 kcal, 0.1 kcal/min), (3) a combination of C12 and Trp (‘C12+Trp’; load: 36 kcal, 0.4 kcal/min), or (4) control (isotonic saline) on fasting plasma glucose, insulin and glucagon concentrations. The choice of nutrient loads was based on our previous studies, which had established sub-maximal effects on gut hormones, and doses were less than those shown to suppress energy intake [11,12,22].

After an overnight fast, participants attended our clinical laboratory in the morning at 8:00 am, and were intubated, via an anaesthetised nostril, with a manometry catheter used for monitoring antropyloroduodenal pressures and duodenal infusion of the treatment solutions [20]. These were infused through a dedicated infusion port located ~14.5 cm beyond the pylorus. Once the catheter was in the correct position [23], and an intravenous cannula placed in an antecubital vein for blood sampling, two baseline blood samples (10 mL) were taken (*t* = −10 min and *t* = 0 min), during phase I of the migrating motor complex (a period of motor quiescence). Infusion of one of the nutrient solutions, or control, was then commenced and continued for 90 min (*t* = 0–90 min). During the infusion, blood samples were collected every 15 min. At *t* = 90 min, the infusion was ceased, the nasoduodenal catheter removed. The participant was offered a cold buffet-meal and instructed to consume as much as they wished until comfortably full (*t* = 90–120 min) [20]. A final blood sample was taken at *t* = 120 min. The intravenous cannula was removed, and the participant was free to leave the laboratory.

### 2.3. Control and Nutrient Treatments

The solutions were prepared as follows: for the C12 solution, we used 5.55 g of food-grade lauric acid (C12:0) (Sigma-Aldrich, Milwaukee, WI, USA), 0.65 g NaOH and 4.5 g NaCl; for the Trp solution, we used 4.07 g crystalline, food-grade L-tryptophan (PureBulk Inc., Roseburg, OR, USA), 0.1 g NaOH and 4.1 g NaCl; for the C12+Trp solution, we used 5.55 g C12 and 4.07g crystalline L-tryptophan, 0.8 g NaOH and 3.8 g NaCl; for the control solution we used 4.9 g NaCl and 0.08 mL NaOH solution (prepared by dissolving 1.75 g NaOH in 250 mL water). All solutions were made to a final volume of 497 mL, were isotonic (~300 mOsm), had a pH of ~7.7-8.1 and were infused at a rate of 3 mL/min, so that 270 mL was the total volume administered in 90 min.

### 2.4. Measurements

#### Plasma Glucose, Insulin and Glucagon Concentrations

Venous blood samples (10 mL) were collected into ice-chilled ethylenediaminetetraacetic acid-treated tubes. Plasma was separated by centrifugation at 3200 rpm for 15 min at 4 °C within 15 min of collection and stored at −80 °C until analysed, as described [20].

Plasma glucose (mmol/L) was measured using a YSI2300 analyser (YSI, Inc., Yellow Springs, OH, USA). Intra- and inter-assay coefficient variations (CVs) were ≤2%.

Plasma insulin (mU/L) was determined by enzyme-linked immunosorbent assay (ELISA, 10-1113, Mercodia, Uppsala, Sweden). Intra- and inter-assay CVs were 2.4% and 9.5%, respectively. The detection limit was 1 mU/L.

Plasma glucagon (pg/mL) was quantified using an RIA (GL-32K, Millipore, Billerica, MA, USA). Intra- and inter-assay CVs were 3.2 and 6.1%, respectively. The detection limit was 20 pg/mL.

### 2.5. Data and Statistical Analyses

Power calculations were used to determine the number of participants, and indicated that *n* = 16 would allow detection of a 15% decrease in energy intake at α = 0.05 with a power of 80% [20]. A secondary calculation indicated that this number would also allow detection of a 0.8 mmol/L difference in plasma glucose.

Baseline (‘0’) values were calculated as the mean of values obtained at *t* = −10 and 0 min. Raw data of plasma glucose, insulin and glucagon concentrations were used to calculate areas under the curve (AUCs), from *t* = 0–90 min, using the trapezoidal rule. The insulin-to-glucose ratio was calculated, using AUCs for insulin and glucose from individual participants, with the following formula: insulin-to-glucose ratio = insulin AUC/glucose AUC. The plasma glucose nadir was determined as the lowest value during the infusion period.

Statistical analyses were performed with SPSS software (version 24.0; SPSS Inc., IBM Corp, Somers, NY, USA). One-factor analysis of variance (ANOVA) was used for analyses of AUCs for glucose and hormone levels and insulin-to-glucose ratio, glucose nadir, and glucose and hormone concentrations at *t* = 120 min (i.e. immediately after the buffet-meal). For all ANOVAs, sphericity was evaluated by Mauchly’s test, and when violated, the adjusted Greenhouse–Geisser *p* value was reported. Normality assumption was met for all outcomes. Post hoc paired comparisons, adjusted for multiple comparisons by Bonferroni correction, were performed where ANOVAs were significant. Plasma glucose and hormone concentrations at *t* = 120 min were compared with concentrations at *t* = 90 min (i.e. before the buffet-meal) using paired *t*-tests. *p* values *<* 0.05 were considered statistically significant, and all data were reported as means ± standard error of the mean (SEM).

## 3. Results

The study treatments were well tolerated. Plasma insulin could not be measured in one participant due to technical problems. There were no significant differences in baseline plasma glucose, insulin or glucagon between study days.

### 3.1. Plasma Glucose

**Figure 1:**
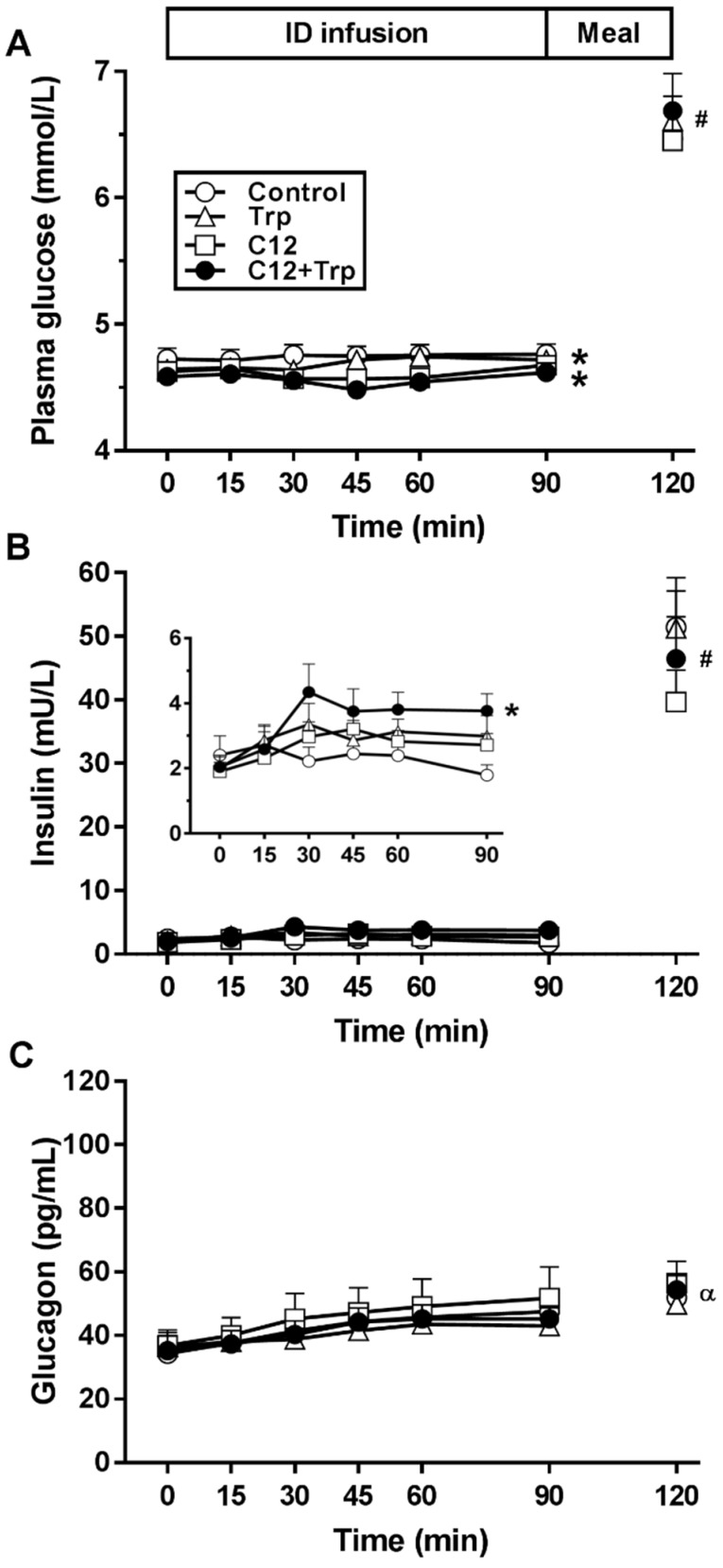
Plasma glucose (**A**), insulin (**B**), and glucagon (**C**) concentrations during duodenal administration of lauric acid (“C12”; 0.3 kcal/min), L-tryptophan (“Trp”; 0.1 kcal/min), a combination of C12 and Trp (“C12+Trp”; 0.4 kcal/min), or isotonic saline (“control”), for 90 min and, at *t* = 120 min, after a buffet-meal. Data (AUCs of glucose and hormone profiles) were analysed using one-way repeated measures ANOVAs, followed, if significant, by post hoc paired comparisons, adjusted for multiple comparisons by Bonferroni’s correction. Post-meal (*t =* 120 min) and pre-meal (*t* = 90 min) values were compared using paired *t*-tests. * *p* < 0.05 vs. control; # *p* < 0.05 all treatments vs corresponding values at *t* = 90 min; α *p* < 0.05 C12+Trp and Trp vs. corresponding values at *t* = 90 min. Data are means ± SEM, *n* = 16, except insulin, *n* = 15.

There was an effect of treatment on glucose AUC (*p* < 0.01) (Table 1, Figure 1A). C12 and C12+Trp, but not Trp, reduced the plasma glucose AUC compared with control (both *p* < 0.05), with no significant differences between C12+Trp and C12 or Trp. There was an effect of treatment on nadir glucose (*p* < 0.05) (Table 1), which was less during C12 and C12+Trp compared with control (all *p* < 0.05), with no significant difference between nutrient treatments.

Following the meal, plasma glucose increased when compared with *t* = 90 min (*p* < 0.05), with no difference between treatments at *t* = 120 min.

### 3.2. Plasma Insulin

There was an effect of treatment on insulin AUC (*p* < 0.05) (Table 1, Figure 1B). C12+Trp, but not C12 or Trp, increased insulin AUC compared with control (*p* < 0.05).

Following the meal, insulin increased when compared with *t* = 90 min (*p* < 0.05), with no difference between treatments at *t* = 120 min.

### 3.3. Insulin-to-Glucose Ratio

There was an effect of treatment on the insulin-to-glucose ratio (*p* < 0.01) (Table 1), which was greater with C12+Trp, but not C12 or Trp, compared with control (*p* < 0.01), with no significant differences between C12+Trp and C12 or Trp.

### 3.4. Plasma Glucagon

Plasma glucagon was not affected by the nutrient treatments (Table 1, Figure 1C). Following the meal, glucagon was increased with Trp and C12+Trp, but not C12 or control, when compared with *t* = 90 min (*p* < 0.05), with no difference between treatments at *t* = 120 min.

## 4. Discussion

This study investigated the effects of intraduodenal administration of C12 and Trp, alone and in combination, on fasting plasma glucose, insulin and glucagon concentrations, in healthy men. Our data demonstrate that intraduodenal infusion of C12 modestly reduces plasma glucose, and that this effect was not further enhanced by its combination with Trp. This observation contrasts with the additive (and possibly synergistic) effects of C12 and Trp on energy intake and some GI hormones, whereby the combination of C12 and Trp, at loads that individually had no effect, substantially reduced energy intake, associated with markedly augmented release of CCK, and suppression of ghrelin, than observed in response to each nutrient alone [20].

Plasma glucose in response to C12 and C12+Trp, but not Trp, was lower compared with control, suggesting that glucose-lowering was driven by C12. The lack of effect of Trp on plasma glucose is consistent with findings from our previous studies with intraduodenal infusion of Trp at a slightly higher load of 0.15 kcal/min [11], or intragastric administration, where Trp at a dose of 3 g alone had no effect on fasting glucose, although it reduced postprandial glucose in both lean and obese individuals [13], possibly via slowing of gastric emptying, which is pivotal to the regulation of postprandial glycaemic control [4,5]. Our findings may appear to contradict results from animal studies. For example, in rats, a dose of 100 mg L-tryptophan/kg body weight was reported to lower fasting blood glucose levels [24]. It is, however, important to recognise that this dose corresponds to ~7 g in a 70 kg person, in contrast to the dose of ~2.2 g administered in the current study. We have previously reported that Trp, when given intraduodenally, at loads of >4 g can induce nausea [11]. Thus, our findings probably do not contradict these earlier findings, which may represent responses to supraphysiological doses, but suggest that Trp, when administered at doses that are well tolerated by humans, and hence more physiological, does not affect fasting glucose concentrations. 

The mechanisms underlying the observed glucose lowering in response to these nutrients are unclear. Because insulin was higher, and the insulin-to-glucose ratio increased, with C12+Trp, but not significantly in response to C12 or Trp alone, glucose lowering by C12+Trp is likely to reflect, at least in part, insulin stimulation. Interestingly, although neither C12 nor Trp alone had any significant effect on insulin, mean levels were higher in response to both nutrients, although only C12 reduced blood glucose. Thus, other factors are likely to also be relevant.

In an original analysis, we observed potent stimulation of GLP-1 by C12 and C12+Trp; in contrast, the effect of Trp was minor and not statistically significant [20]; while the incretin effect of GLP-1, i.e. the glucose-dependent potentiation of insulin secretion [25], may not apply to the current situation, because it requires circulating glucose concentrations of ~7–8 mmol/L [26], other studies have reported that lower glucose concentrations of ~4–5 mmol/L are sufficient [27]. Animal studies indicate that GLP-1 may also affect blood glucose via different mechanisms. GLP-1 receptors are present on neuronal cells in the hepatoportal system and central nervous system, and appear to contribute to the regulation of glucose homeostasis, independent of changes in insulin secretion [28]. Therefore, it is possible that GLP-1 acted in concert with insulin to lead to greater glucose suppression with C12 as well as C12+Trp.

Because there was no differential effect on plasma glucagon, possibly involving suppression of glucagon by GLP-1, particularly during infusion of C12 and C12+Trp, an effect of glucagon to diminish the glucose-lowering effect of insulin can be ruled out.

Some limitations of our study should be recognised. Only one dose of each nutrient was evaluated. Nutrients were administered intraduodenally so that the small intestinal delivery of nutrients was standardised and potential confounding effects of interindividual variations in gastric emptying were avoided, thus, the effects of intragastric or oral administration warrant investigation. There is increasing recognition of interrelations between glucose metabolism, dietary nutrients and the gut microbiome [29], and dietary changes have the capacity to alter the microbiome within 24 hours of introduction [30]. As we investigated the effects of only single doses, and very small amounts, of individual nutrients, our study was not designed to evaluate effects on the microbiome, and we would not expect these nutrients to have a major, if any, influence acutely; however, this warrants investigation in longer-term studies. Moreover, it would be of interest to evaluate whether the composition of the microbiome may influence the magnitude of glucose lowering in response to these nutrients across individuals. Only effects on fasting plasma glucose were evaluated. The study was performed in healthy people, because energy intake represented the primary study outcome. Given our observations of effects on blood glucose lowering in healthy people with good blood glucose control, we would expect more pronounced blood glucose lowering in people with type 2 diabetes with elevated fasting and postprandial blood glucose concentrations.

## 5. Conclusions

The combined intraduodenal administration of C12 and Trp, in healthy men, modestly reduced fasting plasma glucose, an effect most likely driven by C12, and possibly involving both insulin and GLP-1. In light of the observed glucose-lowering effect, studies in healthy participants postprandially and in patients with type 2 diabetes are now warranted. If effective, these nutrients may potentially offer a novel, nutrient-based treatment option for the management of hyperglycaemia in people with type 2 diabetes.

## Figures and Tables

**Table 1:** Plasma glucose, insulin and glucagon concentrations, and insulin-to-glucose ratio during duodenal administration of lauric acid (“C12”; 0.3 kcal/min), L-tryptophan (“Trp”; 0.1 kcal/min), a combination of C12 and Trp (“C12+Trp”; 0.4 kcal/min), or isotonic saline (“control”), for 90 min.

	Control	C12	Trp	C12+Trp	*p* Value
Plasma glucose AUC (mmol/L*min)	426 ± 7	414 ± 5 *	422 ± 4	410 ± 5 *	<0.05
Plasma glucose nadir (mmol/L)	4.7 ± 0.1	4.5 ± 0.1 *	4.6 ± 0.1	4.4 ± 0.1 *	<0.05
Plasma insulin AUC (mU/L*min)	210 ± 35	246 ± 28	266 ± 39	318 ± 47 *	<0.05
Plasma glucagon AUC (pg/mL*min)	3849 ± 457	4146 ± 690	3675 ± 492	3787 ± 375	NS
Insulin-to-glucose ratio	0.5 ± 0.1	0.6 ± 0.1	0.6 ± 0.1	0.8 ± 0.1 ^#^	<0.01

Data are expressed as means ± standard error of the mean (SEM), *n* = 16 for glucose data and glucagon, *n* = 15 for insulin and insulin-to-glucose ratio. Repeated-measures analysis of variance (ANOVA), with treatment as a factor, was used to determine main treatment effects; post hoc comparisons (with Bonferroni correction) were performed when the ANOVA revealed significant effects. * *p* < 0.05 vs. control; ^#^ *p* < 0.01 vs. control; AUC, area under the curve; NS, not significant.
